# Somnal

**Published:** 1891-09-19

**Authors:** 


### THERAPEUTICS.

### SOMNAL.

Somnal is a recently introduced hypnotic, and one which seems to deserve careful clinical investigation. It was introduced about eighteen months ago by Radlauer, of Berlin, and has been tested by Dr. Gilman Thompson, of New York, and by Dr. Frank Woodbury, of Philadelphia, who describes the drug as a colourless liquid resembling chloroform in its appearance and behaviour when added to cold water, in which it forms globules and refuses to mix or dissolve; when shaken with water the mixture is milky, but quickly separates. It is soluble in hot water and alcoholic solutions, and dissolves resinous substances and fats. The odour is faint, not very penetrating or disagreeable, and resembling that of the spirits of nitrous ether or recrystalized chloral. The taste is very pungent, and for administration he finds it needs free dilution; it may be given with whisky or syrup of liquorice. Somnal is inflammable, burning with an alcoholic flame; it does not evaporate quickly, and leaves a greasy stain upon blotting paper. Its specific gravity is greater than water; and it reddens litmus paper slightly.* Dr. Thompson writes† that it is a complex body resulting from the admixture of chloral, alcohol, and urethan. From his five experiments made at the Loomis Laboratory, it appears that:—

1. The ordinary dose of somnal (thirty minims for man) may be given by hypodermic injection to dogs without other effect than drowsiness and slight vertigo and muscular tremor.

2. A dose of one and a-half fluid drachms failed to affect a cat, except in the same manner as dogs.

3. A fatal dose of one and a-half fluid ounces stopped the respiration before the heart, and caused congestion of all the abdominal viscera.

4. The blood pressure in the arteries of a dog is temporarily increased by somnal, soon returning to the normal.

* "Diebetic Gazette," July, 1890.

† "New York Medical Journal," November 29, 1890.

Further, Dr. Thompson states that in the past few months he has given somnal on fifty-four occasions, in doses varying from thirty minims to a drachm, to forty different patients, care being taken to select only those patients who presented well-marked cases of insomnia. The cases were selected also with a view of having as great a variety as possible in the causes of insomnia. The list includes, therefore, insomnia due to rheumatism, phthisis, bronchitis (cough), typhoid delirium, delirium tremens, sciatica, various forms of pelvic pain, neuralgias, &c.

Of the fifty-four instances in which somnal was given, it produced sleep twenty-six times within fifteen minutes, and forty-three times within an hour. In six cases only was it noted as having no effect at all. In a few other instances where it failed to induce sleep it was found to have a very soothing and quieting effect. Sixteen patients slept practically all night after taking half a drachm. Fifteen more slept between three and six hours, and the remainder for briefer intervals. In most of the patients the sleep was natural in character; in only one or two instances did it seem more profound than usual. No after-effects were noticed in any case, with one exception—that of a patient with tuberculosis, who slept seven hours and a-half after a half-drachm dose, and felt depressed on awakening. Most of the patients felt considerably refreshed, many of them decidedly so. There was no disturbance of the stomach or of digestion, with one exception, where a patient with endometritis complained of pain after taking a dose of half a drachm. A patient with delirium tremens had to be quieted by other remedies, and a case of almost maniacal delirium in typhoid fever was unaffected by a dose of forty minims.

CONCLUSIONS.—(1) The effects of somnal are much more striking and certain than those of urethan, and far less depressing than those of chloral.

(2) There is no vertigo or depression after taking somnal, such as may follow the use of sulphonal.

(3) The action of somnal is usually very prompt, and doses of half a drachm, disguised in a little syrup of tolu or whiskey, are always well borne, easily taken, and entirely without deleterious effect.

(4) The drug, in doses of a drachm, is not powerful enough to decidedly control delirium tremens, maniacal delirium, or severe pain.

(5) In doses of thirty or forty minims somnal is a safe and reliable hypnotic for ordinary insomnia.

It is interesting to compare this hypnotic with the far less satisfactory sulphonal, which is certainly more uncertain in its action. Not infrequently patients remain drowsy the following day, even though the sleep of the night has been fitful and unresting. The therapeutic committee, of which Dr. Lauder Brunton is Chairman, appointed by the British Medical Association to determine the relative utility of the different hypnotics, reported that in six out of ten cases, in which twenty grains of sulphonal had been given, disagreeable after-effects were noted; drowsiness next day was noted six times, giddiness four times, and headache and inco-ordination of gait each twice. In four cases, where ten grains had been given, drowsiness was noted once; in five cases, with fifteen grains, drowsiness was noted twice and giddiness twice; with twenty-five grains (four cases), drowsiness was noted twice and giddiness once, and headache once. In seven cases, with thirty to sixty grains, drowsiness was noted four times, giddiness twice, headache twice, inco-ordination of gait and vomiting each once. In some cases prolonged use of the drug seemed to diminish its effect, in others it appeared to have a cumulative effect.

### NEW DRUGS, APPLIANCES, AND THINGS MEDICAL.

[All preparations, appliances, novelties, etc., of which a notice is desired, should be sent for The Editor, to care of The Manager, 140, Strand, London, W.C.]

